# µXRF Elemental Mapping of Bioresorbable Magnesium-Based Implants in Bone

**DOI:** 10.3390/ma9100811

**Published:** 2016-09-30

**Authors:** Anna Turyanskaya, Mirjam Rauwolf, Tilman A. Grünewald, Martin Meischel, Stefanie Stanzl-Tschegg, Jörg F. Löffler, Peter Wobrauschek, Annelie M. Weinberg, Helga C. Lichtenegger, Christina Streli

**Affiliations:** 1 Atominstitut, TU Wien, Stadionallee 2, 1020 Vienna, Austria; mrauwolf@ati.ac.at (M.R.); wobi@ati.ac.at (P.W.); streli@ati.ac.at (C.S.); 2 Institute of Physics and Materials Science, University of Natural Resources and Life Sciences (BOKU), Peter-Jordan-Straße 82, 1190 Vienna, Austria; tilman.gruenewald@boku.ac.at (T.A.G.); martin.meischel@boku.ac.at (M.M.); stefanie.tschegg@boku.ac.at (S.S.-T.); helga.lichtenegger@boku.ac.at (H.C.L.); 3 Laboratory of Metal Physics and Technology, Department of Materials, ETH Zurich, Vladimir-Prelog-Weg 4, 8093 Zurich, Switzerland; joerg.loeffler@mat.ethz.ch; 4 Department of Orthopaedics and Orthopaedic Surgery, Medical University Graz, Auenbruggerplatz 5, 8036 Graz, Austria; annelie.weinberg@t-online.de

**Keywords:** elemental imaging, yttrium, magnesium, biodegradable Mg implants, bone, µXRF

## Abstract

This study investigated the distribution of the elemental constituents of Mg-based implants at various stages of the degradation process in surrounding bone tissue, with a focus on magnesium (Mg), as the main component of the alloy, and yttrium (Y), due to its potential adverse health effects. The measurements were performed on the implant-bearing thin sections of rat bone in a time series of implant degradation between one and 18 months. Micro X-ray fluorescence analysis (μXRF) with a special spectrometer meeting the requirements for the measurements of low-Z elements was used. It was found that the migration and accumulation behaviour of implant degradation products is element-specific. A sharp decrease in Mg was observed in the immediate vicinity of the interface and no specific accumulation or aggregation of Mg in the adjacent bone tissue was detected. By contrast, Y was found to migrate further into the bone over time and to remain in the tissue even after the complete degradation of the implant. Although the nature of Y accumulations must still be clarified, its potential health impact should be considered.

## 1. Introduction

Bioresorbable materials offer a complete rethink of the implant concept and hold great promise in orthopaedic surgery. The rationale for using bioresorbable materials stems from their ability to serve as a load-bearing support for the healing bone, enabling newly-formed healthy tissue to steadily replace the implant and, if possible, even promote tissue formation. Thus, follow-up surgery in order to remove the implant after the tissue has healed is avoided, as well as the risk of problems posed by permanent implants, such as long-term endothelial dysfunction, physical irritation, or chronic inflammatory local reactions [1].

One of the most promising classes of biodegradable materials in load-bearing applications are metal-based alloys, such as those that are Mg-, Fe-, and Zn-based [2,3], due to their superior combination of strength and ductility in comparison to polymers [1]. Novel processing possibilities have also helped to overcome old problems associated with metallic implants, such as uncontrolled degradation and excessive production of gaseous by-products [2].

The implants composed of magnesium-based alloys possess the advantageous characteristics over Fe-based and Zn-based ones, due to the physicomechanical and -chemical properties of magnesium and its role in biochemical reactions [4,5]. Magnesium is one of the most abundant cations in the human organism, it is involved in a number of physiological processes, and up to 85% of body magnesium is stored in bone [6,7]. Additionally, magnesium might serve as an active promoter of new bone formation. For example, Janning et al. demonstrated in a rabbit animal model that an enhanced bone volume adjacent to slowly dissolving magnesium hydroxide cylinders was caused by an enhanced osteoblast activity and a temporarily decreased bone resorption [8]. A recent study by Morawska-Chochol et al. showed that multiphase composite with TCP (tricalcium phosphate) and Mg can stimulate apatite mineralization [9].

Since implant degradation has to be counterbalanced by new tissue formation, in order to maintain mechanical integrity, controlling the degradation rate in the body environment remains the key challenge in current design approaches. The addition of rare earth elements, such as yttrium, can improve the performance of magnesium-based alloys in terms of strength, ductility, and corrosion speed [10]. A study by Pichler et al. demonstrated superior performance of the slower degrading WZ21 alloy, a candidate for use in paediatric orthopaedics [11]. However, yttrium is not normally found in the human body and, therefore, has no known biological role and is regarded as a potentially toxic element, which might unfavourably affect metabolism, especially in children. Yttrium, similar to other rare earth elements, can act as a calcium antagonist. Indeed, yttrium demonstrated inhibitory activity in neuronal calcium ion channels [12]. The inhibitory action of yttrium chloride on the Zn^2+^ influx in rat thymocytes was observed by Takahashi et al. [13]. Feyerabend et al. [14] revealed effects of yttrium on viability of macrophages along with the induction of inflammatory reaction. Toxicological properties of yttrium oxide nanoparticles have been studied in more detail recently, cytotoxicity and genotoxicity were reported in a human embryonic kidney (HEK293) cell line [15], and size-dependant cytotoxicity of Y_2_O_3_ nanoparticles on osteoblasts was observed by Zhou et al. [16].

Evidently, before the material can be introduced into practice, thorough studies of the processes of implant degradation and the mechanisms of migration/incorporation of the implant constituents into bone tissue must be performed in animal models. The non-destructive technique of micro X-ray fluorescence analysis (μXRF) perfectly suits this purpose, allowing the determination of the spatial distribution of major, minor, and trace elements in the sample on the micrometre scale. The method is successfully employed in bone and cartilage research [17,18,19], and usually synchrotron radiation as an excitation source is used for such tasks.

In the present study, we aim to investigate Y (medium-Z element), as a medically-relevant degradation product, along with Mg, the main component of the alloy. Mg is an element of low atomic number, emitting low energy fluorescence photons, which calls for the measuring system with certain characteristics. The µXRF setup at Atominstitut, Vienna, has already proven its suitability for analysing light elements [20,21] and the potential for studying biological samples [22]. In our investigation, we used µXRF to visualize the elemental distribution in bone tissue adjacent to a Mg-based implant at various stages of the degradation process with good spatial resolution. We found that the migration and accumulation behaviour of the degradation products is highly element-specific, with Y showing notably pronounced spreading and accumulation behaviour during implant resorption.

## 2. Results

### 2.1. Laboratory µXRF Analysis 

**Figure 1:**
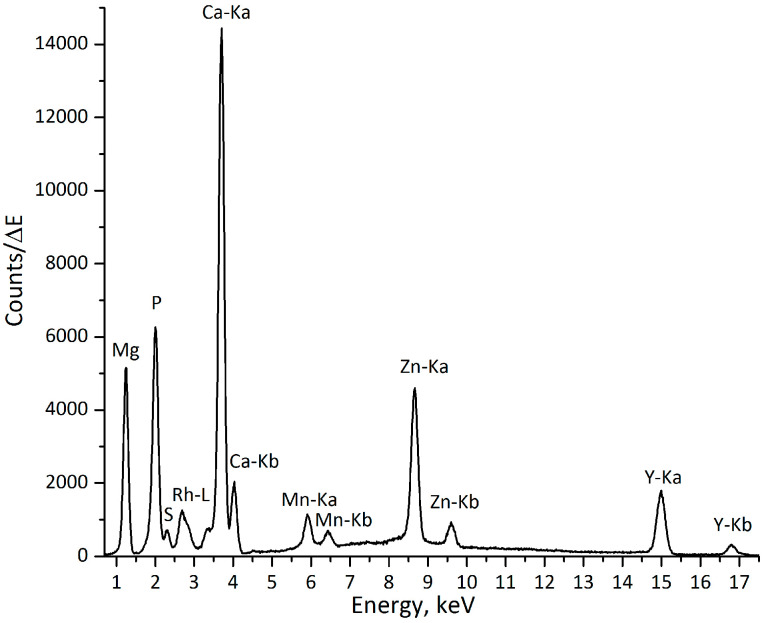
Exemplary spectrum from implant-bone interface (excitation with a Rh-anode X-ray tube), measuring time: 200 s.

The analysis was performed using the laboratory µXRF spectrometer, Atominstitut, Vienna (see Section 4.3). The following elements were identified as implant constituents: magnesium (Mg), calcium (Ca), manganese (Mn), zinc (Zn), and yttrium (Y). The mineralized areas of the bone can be distinguished by the high content of calcium (Ca) and phosphorus (P), which are two of the main constituents of hydroxyapatite (HA, Ca_10_(PO_4_)_6_(OH)_2_). Sulfur (S) is also present in the bone tissue, and less mineralized proteoglycan-containing regions of bone can, therefore, also be identified, as they are rich in sulfur. Figure 1 demonstrates an exemplary XRF spectrum obtained at the interface implant-bone, with both components of the implant (Mg, Mn, Zn, and Y) and bone constituents (P, S, and Ca) being present.

### 2.2. Elemental Mapping

The implant-bearing thin (about 200 µm) sections of rat bone tissue were scanned in raster mode (x-y coordinates). The area of interest in each sample was chosen individually, depending on the morphology of the bone and the state of the implanted material. In the sections, where large parts of the pin remained (one, three, and 12 months dwelling time), scans were performed transverse to the pin. For the samples, where the greatest part of the pin degraded (six, nine, and 18 months dwelling time), the measurements were performed on the former position of the pin, where it had already been replaced by newly-formed bone tissue.

**Figure 2:**
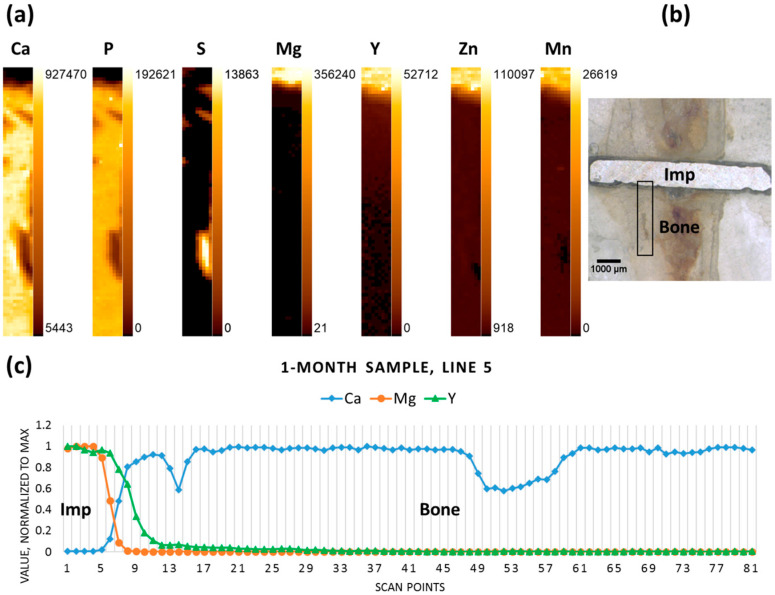
One-month sample (**a**) elemental maps, 9 × 81 pixels—corresponding to 0.4 mm × 4 mm; (**b**) micrograph (obtained with a Wild M8 stereo microscope (Wild Heerbrugg, Heerbrugg, Switzerland)), adapted with permission from Ref. [23]. 2024 Elsevier, the black rectangle outlines the area where the scanning was performed; and (**c**) line scan (line 5); “Imp” denotes the position of the implant, “Bone” denotes the adjacent bone tissue.

The elemental maps were produced for Mg, Mn, Y, P, S, and Ca, based on the data obtained. The maps display the distributions of these elements according to their intensities in the scanned area. Figure 2a shows the elemental maps obtained for the one-month-old sample. The area selected for the scan (Figure 2b) starts at the implant and covers a substantial part of the adjacent bone, allowing the observation of the change in the distribution of the elements of interest in the implant- and bone-containing parts of the scan. For easier comprehension of Mg, Y, and Ca variations a single central longitudinal line from the corresponding elemental maps was selected, the values were normalized to the maximum and the line was plotted (Figure 2c). It can, therefore, be seen for the one-month-old sample, that a sharp decrease in Mg, contrasting with a simultaneous elevation of Ca, indicates the implant-bone interface. As expected, Mg and Y were roughly co-localized, but in the case of Y a less abrupt decrease from the implant towards the proximal bone tissue can be observed.

**Figure 3:**
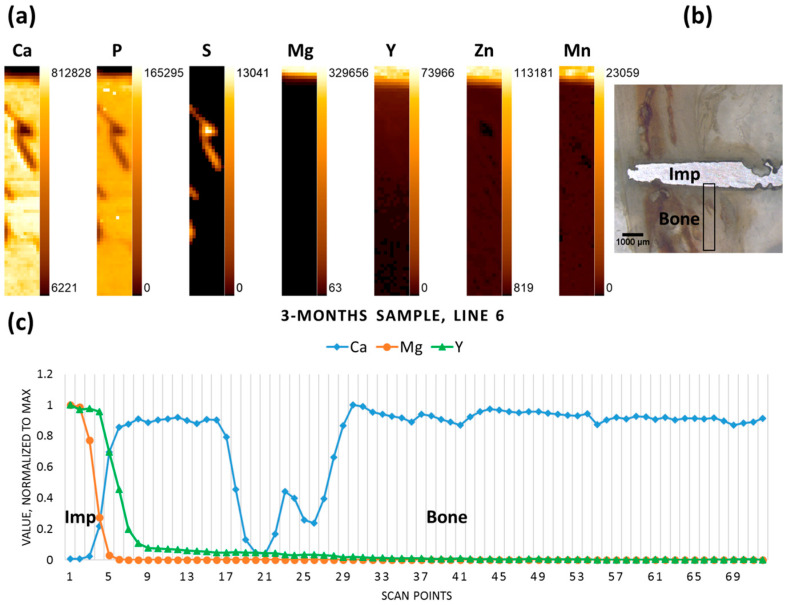
Three-month sample (**a**) elemental maps, 11 × 72 pixels—corresponding to 0.5 mm × 3.55 mm; (**b**) micrograph denoting the scanning area; and (**c**) line scan (line 6).

The three-months-old sample, where degradation had already progressed, yet a large part of the pin remained in the tissue, exhibits similar behaviour for the elements in question, with a drop in Mg and an increase in Ca designating the interface, while Y gradually decreases over some distance from the interface (Figure 3).

**Figure 4:**
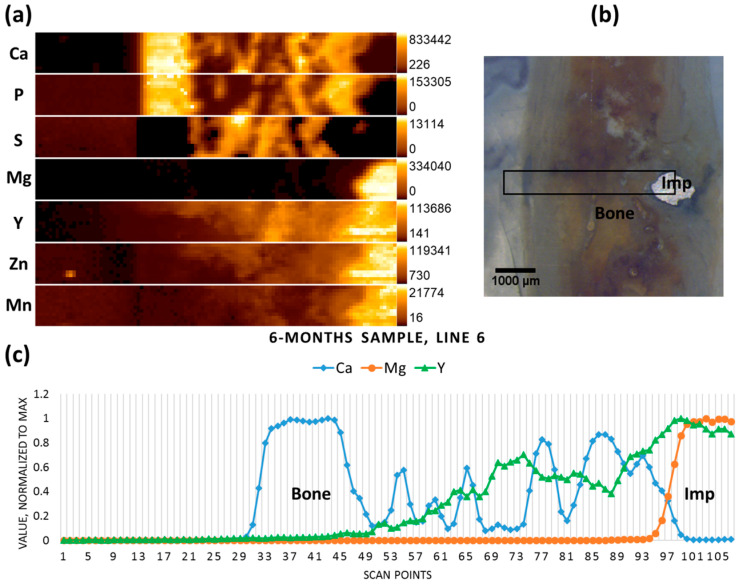
Six-month sample (**a**) elemental maps, 107 × 12 pixels—corresponding to 5.3 mm × 0.55 mm; (**b**) micrograph showing the scanning area; and (**c**) line scan (line 6).

Figure 4b shows the micrograph of the six-month-old sample, where only a small part of the implanted pin remained. The scan performed on the previous position of the pin (including the remaining implant chunk) reveals no specific accumulations of Mg in the newly formed bone tissue, whereas it can be seen that Y has remained on the position of the pin, even in the regions where the implant had already degraded (Figure 4a,c).

**Figure 5:**
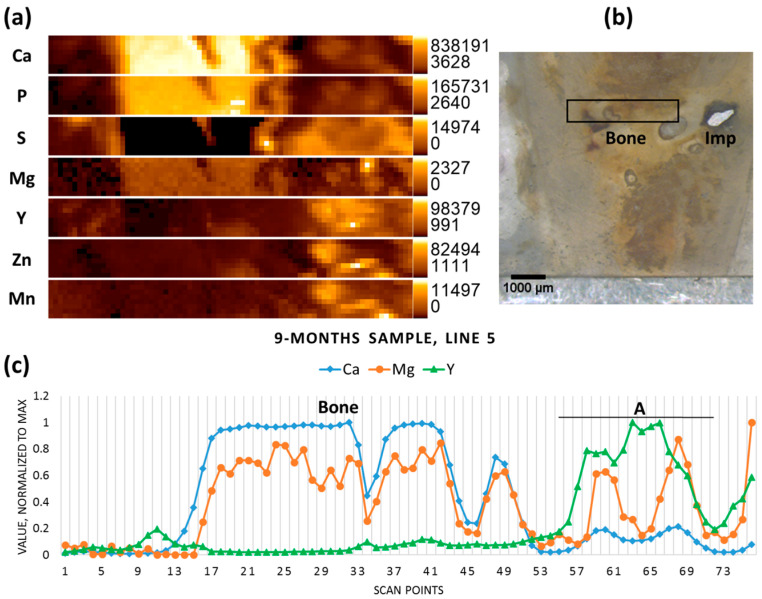
Nine-month sample (**a**) elemental maps, 76 × 8 pixels—corresponding to 3.75 mm × 0.35 mm; (**b**) micrograph showing the scanning area, adapted with permission from Ref. [23]. 2024 Elsevier; and (**c**) line scan (line 5), A—accumulation of Y.

The implanted pin in the nine-month-old sample had mostly degraded, though some metal chunks were still visible; the scanned area lies on the previous position of the implant, already replaced by newly-formed tissue (Figure 5b). No metallic particles could be recognised in this selected area using the microscope, however both the elemental map and the line scan indicate the presence of the minute Mg-containing particles (hot spot on the Mg elemental map), as well as elevated Y (together with the other implant constituents—Mn and Zn) in the right-hand section of the scanned area, which represents less mineralized bone (Figure 5a,c). In the newly-formed mineralized bone tissue (high Ca and P, low to no S) low levels of Mg were co-localized with Ca, as usual in healthy bone.

**Figure 6:**
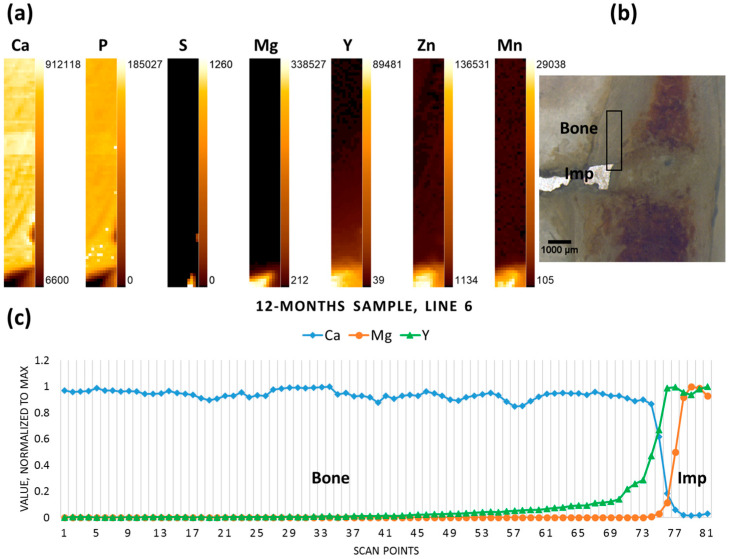
Twelve-month sample (**a**) elemental maps, 11 × 81 pixels—corresponding to 0.5 mm × 4 mm; (**b**) micrograph showing the scanning area, adapted with permission from Ref. [23]. 2024 Elsevier; and (**c**) line scan (line 6).

In contrast to the six- and nine-month samples, the 12-month-old sample contained rather large non-degraded metal pieces of the implant (probably due to the interindividual differences of the animals used). Therefore, the scan was performed in cortical bone transverse to the pin. The implant-bone interface can be discerned due to inverse correlation between Mg and Ca. Again, in contrast to Mg, Y does not demonstrate a sharp drop in the interface area, and is found further away from the implant, having apparently migrated into the bone tissue (Figure 6).

**Figure 7:**
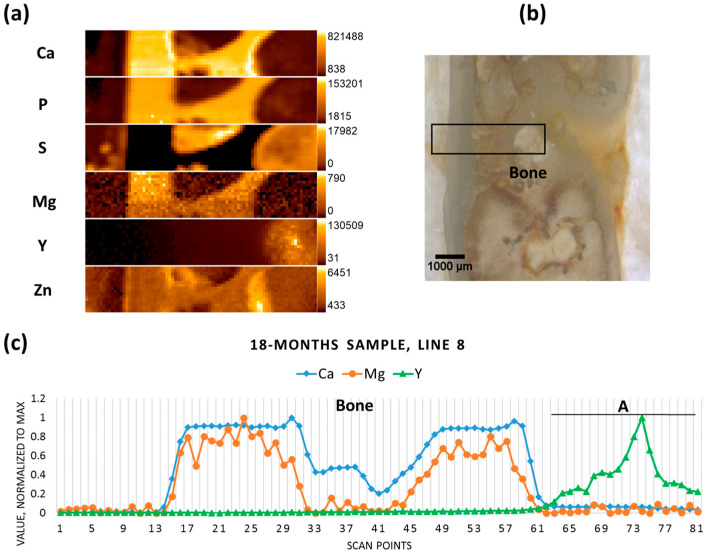
Eighteen-month sample (**a**) elemental maps, 81 × 16 pixels—corresponding to 4 mm × 0.75 mm; (**b**) micrograph showing the scanning area; and (**c**) line scan (line 8).

The degradation process in the 18-months-old sample was on the verge of completion, and no metal pieces could be observed with the microscope. The scan was performed on the previous position of the implant (Figure 7b). As can be seen from the resultant elemental maps, Mg was present at low content in the calcified area of the bone, as usual in healthy bone (Figure 7a). Yttrium, however, was accumulated in the non-mineralized area in the bone marrow and its content decreased towards the corticalis (Figure 7c). No Mn was detected in the sample, and low levels of Zn were mainly found in calcified tissue, which might depict the natural distribution of the element. It should also be mentioned that, in the case of Zn, the peak count rate of 1000 in the non-calcified region must be considered as the background, in contrast to the calcified zone. The elevated spectral background in the non-calcified zone is due to the high scatter contribution of sulfur and organic components of the non-mineralized matrix.

In summary, while Mg content decreased rather sharply at the bone-implant interface (over 99% within ca. 300 µm), and only a very low Mg signal was found in the newly formed bone, Y migrated further into the tissue. The estimation of Y migration is based on the spectral information. In the selected line scan the position in the bone adjacent to the bone-implant interface was defined (the value for Ca is close to 1) and taken as a starting point. The last point on the line scan, for which the Y peak can be observed and distinguished from the spectral background, would mark the migration front line. The distances over which Y migrated and could be detected are listed in Table 1. It is obvious that the distance increased with the dwelling time.

It should be noted, however, that the spatial resolution and information depth differ for each element, and Y can be excited and detected at deeper layers in the sample, in contrast to Ca and Mg (information depth of Mg in bone: 2.3 µm; Ca in bone: 25.7 µm; and of Y in bone: 354 µm (calculated with the X-ray Utilities application)). Therefore, the position of potential Y accumulations (as well as the position of the implant interface) can only be defined approximately.

## 3. Discussion

By means of µXRF analysis we were able to visualize the distribution of the elements of interest in bone tissue, with a focus on Mg and Y. The degradation process is not gradual, and interindividual variation in response to the implant should also be taken into account. Elemental maps and extracted information in the form of line scans showed a sharp decrease in Mg in the immediate vicinity of the interface and no specific accumulation or aggregation of Mg in the adjacent bone tissue in all of the investigated samples. It can, therefore, be assumed that Mg released during the degradation process is steadily included into the metabolic processes. This agrees with recent investigations where local Mg content was probed by µXRF with even higher resolution at the synchrotron, and no Mg accumulation was found at the interface of the WZ21 implant studied, but only around blood vessels, which are beyond the spatial resolution of the present study [24]. The correlation of Mg with Ca might be ascribed to the natural distribution, but there could also be a contribution by secondary excitation of Mg by Ca in highly-mineralized regions of bone. However, it should be admitted, that the analysis of elements with low atomic number is still challenging, and natural concentrations of Mg might lie beyond the detection limits.

In all maps we found a tendency for Y to migrate deeper into the bone tissue than Mg. The distance from the implant rose with the increased dwelling time of the implant in the bone tissue, indicating a progressive distribution and accumulation of Y in bone further away from the implant site. Since Y could also be detected at the former implant position after 18 months, when the implant had already completely degraded, its accumulation has quite a permanent character.

It is known from the literature that Y has a tendency to accumulate in soft tissues, such as liver, spleen, and kidney, and in bone, although the reports about the deposition in bone are conflicting. It was found that small Y particles injected intravenously in rabbits went chiefly to the bone and bone marrow [25]. Intravenous injection in rhesus monkeys after four hours resulted in Y accumulation in the epiphyseal plate and periosteum of the femur, but not in bone marrow [26]. Appelgren et al. observed high concentration of Y in ossification zones and in periosteal and endosteal layers in mice one hour after injection [27]. Herring et al. demonstrated the preferential binding of Y by quiescent and resorbing bone surfaces [28], and it was further shown in vitro that bone sialoprotein might be involved in the mechanism of Y binding [29]. It should be noticed, however, that the route of administration (injection) and the short-term exposure to Y in the above-mentioned studies do not allow to fully extend them to the specific case of bioresorbable implanted material, where the mechanism of accumulation (and binding) might be different. With regard to Y-containing alloys it should be remarked that, in corrosion tests, yttrium migrates to the metal surface of the alloy and is oxidized; therefore, yttrium oxide (Y_2_O_3_) will accumulate in the degradation layer [10]. It could be assumed that the formation of soluble yttrium salts will be taking place at the degradation site, however, further thorough investigations of degradation processes involved are warranted.

Manganese, although it is a minor component of the alloy, should also be mentioned with respect to its physiological and putative toxicological role. Recently, influence of manganese on nervous system and Mn-induced Parkinsonism has attracted much attention [30]. It is suggested that bone tissue substantially accumulates Mn, and during the lifetime it could be released slowly, serving as an internal source of Mn exposure [31]. In our findings, Mn did not demonstrate any specific accumulations, probably due to its low content in the alloy.

Therefore, further investigations are needed, e.g., using confocal µXRF (also synchrotron radiation-induced µXRF) imaging to identify the distribution of elements in different depth layers; synchrotron XRF tomography to determine the migration of Y in the bone volume, or by X-ray absorption spectroscopy to define the Y compounds in bone tissue.

## 4. Materials and Methods

### 4.1. Implant Material

The implant material used for this study was a crystalline magnesium rare-earth-containing WZ21 alloy (detailed description of the alloy can be found elsewhere [5]) with a composition of 2 wt % Y, 1 wt % Zn, 0.25 wt % Ca, 0.15 wt % Mn, and 96.4 wt % Mg. The grain size was determined to be 7 µm. Further information can be found in the literature [32,33]. The implant material was manufactured to pins of 1.6 mm in diameter and 7 mm in length.

### 4.2. Bone Samples

The samples for this investigation (one per dwelling time) were obtained from male Sprague Dawley rat femurs that had been implanted with WZ21 pins at the age of five weeks and were monitored by micro-computed tomography and histological investigations during the course of healing [5]. The interfacial mechanical properties of this kind of alloys, as well as details regarding the design of the experiment, can be found in [33,34]. The experiments were authorized by the Austrian Ministry of Research and Science (Vienna, Austria) under accreditation number BMWF-66.010/0087-II/3b/2011. In this study, six samples with implant dwelling times of 1, 3, 6, 9, 12, and 18 months were used as longitudinal sections embedded in PMMA resin (Technovit 7100, Materials 2016, 9, 811 9 of 11 Heraeus Kulzer, Werheim, Germany). Part of these samples had been used in a previous study to investigate the bone nanostructure with small-angle X-ray scattering [23]. As described in [23], the sections were ground to a thickness of approx. 200 μm using a Struers Planopol-3 (Struers, Ballerup, Denmark) polishing machine with #200 sanding paper.

Microscopic images were obtained using a Wild M8 (Wild Heerbrugg, Heerbrugg, Switzerland) stereo microscope with a “PixeLink PL-B686CF” (Ottawa, ON, Canada) camera system to obtain images with 6× and 25× magnification.

### 4.3. X-ray Fluorescence Analysis

The μXRF setup at Atominstitut (ATI) was designed in-house and is suited for the detection of a wide range of elements, which was essential for this experiment, as both elements of low atomic number (Mg) and medium atomic number (Y) elements were of interest. The detailed description of the setup can be found in previous publications [35,36]. Excellent excitation conditions for the measurements of light and heavy elements are provided by a Rh-anode low power tube (20 W). The Rh tube emits Rh-K line (20.3 keV) radiation which excites Y, but it also emits Rh-L line (2.7 keV) radiation, which excites Mg (Kα = 1.25 keV) excellently. As the setup is in vacuum, the low-energy radiation is not absorbed by air. The following tube settings were employed in the experiment: the voltage was 50.0 kV, and the current was 0.4 mA. The system features a 30 mm^2^ Si(Li) detector with an ultrathin polymer window (UTW), and is LN_2_-cooled. The UTW allows the efficient measurement of the low energy fluorescence radiation from Mg. Excitation and detection conditions, together with the operation under vacuum, facilitates analysis of low atomic number elements, e.g., magnesium. A polycapillary optic (full lens) was used to focus the primary beam on the sample surface; the beam size determined for Cu-Kα was 50 µm × 50 µm.

The measurement conditions were the same for all of the scans. The scan area was defined by determining four corner points in XY coordinates (controlled by a built-in optical microscope with a charge-coupled device camera), the step size was set to 50 µm. The measurement time was 200 s per point.

The data analysis was performed using the QXAS-AXIL software package (IAEA Laboratories Seibersdorf, Seibersdorf, Austria) [37]. The elements of interest were included in the fitting model: Mg, P, S, Ca, Mn, Zn, and Y. The elemental maps were created with X-Ray Lab software (X-ray Lab, Atominstitut, Vienna, Austria). The line scans represent the plotted central line (lengthwise) throughout the scan (data normalized to the maximum value) allowing for easier comprehension of the changes in distributions of elements (prepared using Microsoft Excel 2016). The fluorescence spectrum was plotted using Origin (version 8.6, OriginLab Corporation, Northampton, MA, USA).

## 5. Conclusions 

µXRF was successfully used to investigate the elemental distribution of metals from bioresorbable implants in rats using a special spectrometer suitable for the detection of light elements. Elemental maps indicate a sharp decrease in Mg in the immediate vicinity of the interface and no specific accumulation or aggregation of Mg in the adjacent bone tissue for all of the investigated samples. It can be assumed that Mg released during the degradation process is steadily included into the metabolic processes. By contrast, Y migrated into the bone matrix further away from the interface, with the spread increasing over time. The accumulation seemed to be of a permanent character, since Y can be detected on the position of the implant after 18 months. The nature of the Y accumulations is not clear, it could be due to formation of particles too small to be observed by a conventional microscope, or due to binding to biomolecules, such as glycoproteins. In any case, this should be regarded with great care due to the known toxicity of Y.

## Figures and Tables

**Table 1:** Estimation of Yttrium migration into the bone.

Duration of Stay in Bone	Distance from Interface
1 month	950 µm
3 months	1400 µm
12 months	1700 µm
