# Correction: Turyanskaya et al. µXRF Elemental Mapping of Bioresorbable Magnesium-Based Implants in Bone. *Materials* 2016, *9*, 811

**DOI:** 10.3390/ma18194452

**Published:** 2025-09-24

**Authors:** Anna Turyanskaya, Mirjam Rauwolf, Tilman A. Grünewald, Martin Meischel, Stefanie Stanzl-Tschegg, Jörg F. Löffler, Peter Wobrauschek, Annelie M. Weinberg, Helga C. Lichtenegger, Christina Streli

**Affiliations:** 1 Atominstitut, TU Wien, Stadionallee 2, 1020 Vienna, Austria; mrauwolf@ati.ac.at (M.R.); wobi@ati.ac.at (P.W.); streli@ati.ac.at (C.S.); 2 Institute of Physics and Materials Science, University of Natural Resources and Life Sciences (BOKU), Peter-Jordan-Straße 82, 1190 Vienna, Austria; tilman.gruenewald@boku.ac.at (T.A.G.); martin.meischel@boku.ac.at (M.M.); stefanie.tschegg@boku.ac.at (S.S.-T.); helga.lichtenegger@boku.ac.at (H.C.L.); 3 Laboratory of Metal Physics and Technology, Department of Materials, ETH Zurich, Vladimir-Prelog-Weg 4, 8093 Zurich, Switzerland; joerg.loeffler@mat.ethz.ch; 4 Department of Orthopaedics and Orthopaedic Surgery, Medical University Graz, Auenbruggerplatz 5, 8036 Graz, Austria; annelie.weinberg@t-online.de

**Missing Citation**

In the original publication [1], “Grünewald, T.A.; Ogier, A.; Akbarzadeh, J.; Meischel, M.; Peterlik, H.; Stanzl-Tschegg, S.; Löffler, J.F.; Weinberg, A.M.; Lichtenegger, H.C. Reaction of bone nanostructure to a biodegrading Magnesium WZ21 implant—A scanning small-angle X-ray scattering time study. *Acta Biomater.* **2016**, *31*, 448–457.” was not cited. The citation has now been inserted as reference [23] in the captions of Figures 2, 5, 6, Section 4.2. Bone Samples, Paragraph 1, and should read as follows:

**Figure 2.** One-month sample (**a**) elemental maps, 9 × 81 pixels—corresponding to 0.4 mm × 4 mm; (**b**) micrograph (obtained with a Wild M8 stereo microscope (Wild Heerbrugg, Heerbrugg, Switzerland)), adapted with permission from Ref. [23]. 2024 Elsevier, the black rectangle outlines the area where the scanning was performed; and (**c**) line scan (line 5); “Imp” denotes the position of the implant, “Bone” denotes the adjacent bone tissue.

**Figure 5.** Nine-month sample (**a**) elemental maps, 76 × 8 pixels—corresponding to 3.75 mm × 0.35 mm; (**b**) micrograph showing the scanning area, adapted with permission from Ref. [23]. 2024 Elsevier; and (**c**) line scan (line 5), A—accumulation of Y.

**Figure 6.** Twelve-month sample (**a**) elemental maps, 11 × 81 pixels—corresponding to 0.5 mm × 4 mm; (**b**) micrograph showing the scanning area, adapted with permission from Ref. [23]. 2024 Elsevier; and (**c**) line scan (line 6).

- *4.2. Bone Samples*

The samples for this investigation (one per dwelling time) were obtained from male Sprague Dawley rat femurs that had been implanted with WZ21 pins at the age of five weeks and were monitored by micro-computed tomography and histological investigations during the course of healing [5]. The interfacial mechanical properties of this kind of alloys, as well as details regarding the design of the experiment, can be found in [33,34]. The experiments were authorized by the Austrian Ministry of Research and Science (Vienna, Austria) under accreditation number BMWF-66.010/0087-II/3b/2011. In this study, six samples with implant dwelling times of 1, 3, 6, 9, 12, and 18 months were used as longitudinal sections embedded in PMMA resin (Technovit 7100, Materials 2016, 9, 811 9 of 11 Heraeus Kulzer, Werheim, Germany). Part of these samples had been used in a previous study to investigate the bone nanostructure with small-angle X-ray scattering [23]. As described in [23], the sections were ground to a thickness of approx. 200 μm using a Struers Planopol-3 (Struers, Ballerup, Denmark) polishing machine with #200 sanding paper.

With this correction, the order of some references has been adjusted accordingly.

**Error in Author Contributions**

In the original publication [1], there were errors in the Author Contribution statement. The correct Author Contribution statement appears below.

**Author Contributions:** A.T., M.R., T.A.G., H.C.L., S.S.-T., P.W., C.S., A.M.W. and J.F.L. conceived and designed the experiments; T.A.G., M.M., J.F.L. and A.M.W. prepared the samples; A.T. and M.R. performed the experiments; A.T. analyzed the data; A.T., M.R., T.A.G., M.M., S.S.-T., P.W., H.C.L. and C.S. interpreted the data; A.T. drafted the manuscript. All authors have read and agreed to the published version of the manuscript.

**Removal of the Telephone Number**

In the original publication [1], the corresponding author’s telephone number is no longer in use and has therefore been removed.

The authors state that the scientific conclusions are unaffected. This correction was approved by the Academic Editor. The original publication has also been updated.
